# Spermatozoa Survival in Egg Yolk-Based and Soybean-Based Extenders at Ambient and Chilling Temperature in Domestic Turkeys *(Meleagris gallopavo)*

**DOI:** 10.3390/ani12050648

**Published:** 2022-03-03

**Authors:** Isa Mohammed Alkali, Suleiman Omeiza Asuku, Martina Colombo, Muhammad Modu Bukar, Mohammed Ahmed Waziri, Gaia Cecilia Luvoni

**Affiliations:** 1 Department of Theriogenology, Faculty of Veterinary Medicine, University of Maiduguri, Maiduguri 600230, Nigeria; suleimanomeiza06@gmail.com (S.O.A.); mmbukar@unimaid.edu.ng (M.M.B.); mohwaz@unimaid.edu.ng (M.A.W.); 2 Dipartimento di Medicina Veterinaria e Scienze Animali, Università degli Studi di Milano, 26900 Lodi, Italy; martina.colombo@unimi.it (M.C.); cecilia.luvoni@unimi.it (G.C.L.)

**Keywords:** domestic turkey, peacock, spermatozoa, soybean, egg yolk, extender, L-ascorbic acid, ambient temperature, chilling temperature

## Abstract

**Simple Summary:**

Hunting pressure and loss of habitat exacerbated by climate change have led to a decline in the number of many galliform species, especially those found in the wild. One such species is the Congo peacock, which has been classified as vulnerable. A domestic galliform species such as the domestic turkey can conceivably be used as a model to research and understand reproduction patterns of related wild and endangered species. The collection, preservation and use of the male gametes for assisted reproductive techniques are key to the successful breeding of such species. This study was aimed at developing a suitable semen extender using two extender formulations (egg yolk-based and soybean-based with or without L-ascorbic acid supplementation) and two storage protocols (ambient temperature and chilled). The results show that both extender formulations preserved turkey semen viability for up to 6 h at ambient temperature and up to 24 h at the chilling temperature. In conclusion, the two extenders were similar with regard to semen quality parameters, and L-ascorbic acid supplementation of the turkey semen extenders improved semen quality during liquid storage.

**Abstract:**

Populations of many galliform species have declined mainly due to habitat loss and over-hunting, notably the Congo peacock, which has been classified as a vulnerable species by the International Union for Conservation of Nature (IUCN). The domestic turkey, being a species of least concern, which has been reported to be closely related to peacocks, could serve as a model for the optimization of assisted reproductive technologies for the Congo peacock. This study was aimed at developing a suitable turkey semen extender for artificial insemination in field conditions. Semen was collected using the dorso-abdominal massage technique from seven turkey toms and analyzed. Ejaculates with >70% motility and >80% live spermatozoa were pooled and divided into four aliquots (four treatments). Each of the four treatments was extended in a soybean-based extender or an egg yolk-based extender, with or without L-ascorbic acid. Two liquid preservation protocols (ambient temperature (35 °C) and chilled (4 °C)) were employed, and quality parameters including motility, viability and morphology were evaluated. The results show that the two extenders were similar with regard to semen quality parameters, and L-ascorbic acid supplementation of the turkey semen extenders improved semen quality during liquid storage.

## 1. Introduction

Populations of many galliform species have declined mainly due to habitat loss and over-hunting [1], including those in captivity [2]. Notably, the Congo peacock (*Afropavo congensis*), which inhabits the forested areas of the Democratic Republic of the Congo, has been classified as a vulnerable species by the International Union for Conservation of Nature (IUCN) [3]. This was further compounded by climate change and growing armed conflicts in sub-Saharan Africa which could push more species to the IUCN red list [4]. However, there is a general paucity of information in the literature on galliform research, and very few studies are focused on their conservation [1]. Saini et al. [5] reported that both sequence and phylogenic analysis confirmed that peacocks are more related to turkeys than they are to chickens. Therefore, it is conceivable to use domesticated galliforms such as the domestic turkey (*Meleagris gallopavo*), being a species of least concern, for research and optimization of assisted reproductive technologies (ARTs), which may serve as a model for vulnerable species such as the Congo peacock and perhaps other wild galliforms.

Good-quality semen is the prerequisite to a successful artificial insemination, which is the global best practice for turkey breeding [6]. Turkey semen is highly concentrated and rapidly deteriorates in vitro [7,8]. This could be associated with the unique nature of turkey seminal plasma which is composed of both proteins and proteinases [9]. It has been reported that this negative attribute of turkey semen, when compared with other galliforms, may be due to the deleterious effects of some seminal plasma proteins which, in some instances, have been related to a condition of low semen quality called yellow semen syndrome [10,11]. In addition, preserving and extending turkey semen beyond six hours without a significant loss of quality has not yet been achieved, especially at higher ambient temperatures [12,13,14,15]. Thus, developing an extender that would dilute and maintain the fertilizing capacity of turkey semen at ambient temperature for 24 h and beyond will permit ARTs in field conditions [16].

The existing poultry semen extenders are mostly minor modifications of the protocols developed in the last four decades [13,14,17]. The common constituents considered in these poultry diluents are buffered salt to maintain pH and osmolarity as well as sources of energy for sperm metabolism [7]. Turkey semen thrives better in an acidic medium with a pH between 6.5 and 6.8 [17] and osmolarity between 250 and 330 Osm [18,19]. Constant aeration is essential for maintaining the viability of turkey semen during liquid preservation [20,21]. However, even when these conditions are met, commercially acceptable fertility rates are obtained only for a few hours of preservation [12].

Storing extended semen at reduced temperature helps to extend spermatozoan viability by slowing metabolism and inhibiting bacterial growth, thence lowering the accumulation of metabolic byproducts [22,23,24]. The beneficial positive effect of chicken egg yolk as a non-permeable cryoprotectant in both chilling and sub-zero temperatures has been reported [25,26]. It has been conventionally used in commercial semen extenders to provide sperm membrane protection [27]. However, it has been reported that egg yolk supplementation of avian semen has a negative effect on fertility [28], while according to some authors, a lower concentration of egg yolk did not significantly affect both motility and fertility [29]. Recent findings revealed the positive role of low-density lipoproteins (LDL) found in egg yolk on spermatozoan membrane protection. These LDL rapidly bind and thus neutralize seminal plasma proteins which have been incriminated in causing the rapid loss of turkey semen quality [30,31]. In addition, the use of egg yolk in freezing extenders of Indian red jungle fowl improved semen quality and fertility [32]. Similarly, egg yolk plasma which is obtained after eliminating the larger macromolecules and mainly consists of LDL has been reported to improve rooster sperm quality, fertility and hatchability [31].

Similarly, soybean contains a large proportion of LDL called soybean lecithin, similar to egg yolk lecithin, and fatty acids such as stearic, oleic and palmitic acid which have membrane protection potential during cryopreservation and liquid storage of semen [33,34,35]. Being plant based, soybean has been preferred to replace the conventional egg yolk due to the obvious risk of transmitting pathogenic microorganisms or hormonal substances that may negatively affect semen quality when using animal-based products [33,36]. Similarly, international restrictions on potential sources of exotic infectious diseases could limit the transportation of semen extended using egg yolk [35,37].

Generally, poultry semen contains a variety of natural antioxidants including vitamin E, vitamin C and glutathione, which prevent reactive oxygen species (ROS) from damaging the spermatozoan membrane [38]. However, semen dilution could reduce this ability [39]. Bréque et al. [38] reported that, in avian species, vitamin C may be the prime mover in the complex antioxidant system of sperm storage tubules of the oviduct; thus, we hypothesized that in vitro supplementation of turkey semen with L-ascorbic acid may improve its storage quality.

In order to promote ARTs in field conditions of the tropics, it is pertinent to develop an extender composition that will be suitable for preserving semen at ambient temperature, especially in resource-deficient countries such as those found in sub-Saharan Africa. This study was therefore designed to evaluate the effect of soybean- and egg yolk-based extenders on the liquid storage quality of turkey semen, and to determine the effect of L-ascorbic acid supplementation on turkey semen maintained at ambient and chilling temperatures.

## 2. Materials and Methods

### 2.1. Animals

This study was conducted at the Artificial Insemination Laboratory, Department of Theriogenology, University of Maiduguri, Nigeria. Seven apparently healthy (with no observable signs of disease or ill health) turkey toms and two turkey hens weighing between 8 and 15 kg were procured from a local poultry market in Maiduguri (Nigeria) for the study. The toms (7–10 months) were managed intensively (in an enclosed hall space with windows, a source of ventilation and light) in individual cage enclosures of 3 ft × 3 ft × 3 ft dimension and exposed to at least 18 h of light per day throughout the study period. The hens were kept in adjacent cages to stimulate the toms. Animals were fed daily with commercial feed containing 21% crude protein and drinking water ad libitum. They were treated for both external and internal parasites using oral ivermectin solution (Kepromec^®^, Devender, The Netherlands) and then allowed to acclimatize for two weeks prior to the semen collection procedure. The toms were conditioned and trained to respond to the semen collection procedure using the dorso-abdominal massage technique as described by Burrows and Quinn [40]. Feathers and danders around the vent were trimmed to facilitate collection of clean ejaculates.

The university ethical committee for the use of animals in research classified semen collection as part of routine management procedures, and we duly adhered to the standard management guidelines stipulated.

### 2.2. Experimental Design

Semen was collected from each tom between 7:00 and 9:00 twice weekly as recommended by Noirault et al. [41] and preliminarily analyzed to evaluate baseline semen characteristics and establish a spermiogram of the study subjects. Each ejaculate was analyzed independently. Then, ejaculates were collected for the experiment from each tom and rapidly evaluated for motility and viability. Ejaculates with >70% motility and >80% live spermatozoa were pooled and used for the experiment. Each pooled semen (with at least four ejaculates) was divided into four aliquots (treatments). Each of the four treatments was extended in an egg yolk-based extender (EYR) or soybean-based extender (SBR), with or without L-ascorbic acid supplementation. Therefore, the four treatments were EYR, SBR, EYR+L-ascorbic acid and SBR+L-ascorbic acid. The control was kept as such, without any dilution. The final sperm concentration in each aliquot was 1 × 10^9^ mL^−1^. Then, each of the four treatments was further divided into two. The first batch was kept in partially open 2 mL Eppendorf tubes at ambient temperature (35 °C) using a water bath (Memmert waterbath WNB, Schwabach, Germany) to stabilize the temperature and assessed for semen quality immediately after extension and hourly for 7 h. The second batch was kept in partially open 2 mL Eppendorf tubes at 4 °C in a refrigerator (Haier Thermocool Refrigerator HR 147 S 130L, Hong Kong, China) and assessed for semen quality at 6, 12 and 24 h. The whole experiment was repeated ten times.

### 2.3. Extender Preparation

All chemicals and reagents were purchased from Avantor BDH chemicals (Leicestershire, UK), unless otherwise stated.

Modified Ringer’s solution (R) was prepared by dissolving sodium chloride 120 mM (Sigma-Aldrich Chemical Company, St. Louis, MO, USA), potassium chloride 5 mM, potassium dihydrogen phosphate 10 mM, magnesium phosphate heptahydrate 5 mM and tris hydrochloride 1 mM (Sigma-Aldrich Chemical Company, St. Louis, MO, USA) in double distilled water. Glucose (1%) and a penicillin G and streptomycin combination (1 g L^−1^) were added, and the solution was filtered using Whatman sterile syringe filters (0.2 µm pore size). The soybean-based extender (SBR) contained R and 1% soybean milk, while the egg yolk-based extender (EYR) contained R and 5% egg yolk plasma. Each was supplemented with 20 µg mL^−1^ L-ascorbic acid (Sigma-Aldrich Chemical Company, St. Louis, MO, USA). The pH was adjusted with normal hydrochloric acid to 6.8–7.

### 2.4. Soybean Extraction Method

The soybean was prepared according to the method described by Singh et al. [42] with slight modification. An amount of 2 g of soybean was washed and soaked in 100 mL distilled water and then boiled for 30 min. After boiling, the water was discarded, and the whole soybean was washed again and soaked in 0.25% sodium trioxocarbonate for 30 min. The solution was discarded afterward, and the soybean was blended with 100 mL of distilled water. Soybean milk was extracted by filtration through a sieve and double centrifuged, and the supernatant was used for the study.

### 2.5. Egg Yolk Plasma Preparation

Egg yolk extraction was performed according to Ali and Wu [43] with modification. Briefly, freshly laid chicken eggs were collected from the University of Maiduguri farm, washed with running tap water and disinfected with 70% ethanol. Eggs were manually broken, most of the albumen was removed and the egg yolk was placed on filter paper (Whatman No. 1) and rolled until all the albumen was removed. A sterile syringe was used to aspirate the yolk after piercing through the yolk sac. An equal volume of the yolk and normal saline (120 mM) was placed in sterile tubes and centrifuged twice at 2000× *g* for 45 min. The supernatant was used for extender preparation. All extraction processes were conducted at room temperature.

### 2.6. Semen Collection

Semen was collected using the abdominal massage technique described by Burrows and Quinn [40]. Briefly, the collector restrained the turkey tom by its thighs with one hand while the other hand held the collecting tube. An assistant placed the turkey tom between his two legs, massaged the soft part of the abdomen with his left hand to protrude the lateral phallic bodies (tumescence) while simultaneously passing the palm of his right hand gently over the vent and pushed the tail back over the tom’s back with the heel of the right hand. The assistant maintained pressure on the tail head until the thumb and index finger of the left hand were in position to gently squeeze behind the phallus. The semen was then milked and collected in a graduated glass tube. Collections were carried out between the months of May and August.

### 2.7. Semen Evaluation

#### 2.7.1. Motility

Motility evaluation was performed by placing one drop of the semen on a glass slide, diluting it with an extender/normal saline and covering it with a glass cover slip. The sperm motility was estimated subjectively as reported earlier [44] by at least three operators. Motility was expressed as a percentage of motile spermatozoa with moderate to rapid progressive movement [45].

#### 2.7.2. Concentration

The sperm concentration was determined as described in the literature [46] using a Neubauer chamber. Semen was diluted (1:200) with 4% formal saline before charging the Neubauer chamber.

#### 2.7.3. Viability

Sperm viability (live/dead ratio) and morphology were determined using eosin-nigrosin-stained slides [47,48]. Briefly, an eosin-nigrosin stain smear was created by placing a drop of 1:2 solution of eosin-nigrosin on a glass slide followed by a drop of semen and gentle mixing. A smear of the mixture was allowed to air dry and viewed with immersion oil at (×1000) magnification. A differential count on a sample of 200 total spermatozoa from different microscopic fields was performed and classified in percentage of the unstained spermatozoa as “Live“ and the stained or partially stained spermatozoa as “Dead“ (Figure S1).

#### 2.7.4. Morphology

Sperm morphology was evaluated from the eosin-nigrosin-stained smears. A total of 200 spermatozoa were counted, classified and expressed as a percentage of abnormal against normal spermatozoa. Various morphological defects were also classified and grouped into four categories, viz., acrosomal (detachment and swelling of the acrosome), head (bent head, swollen head, looped head and detached head), mid-piece (swollen, bent or thickened mid-piece and cytoplasmic droplet) or tail defects (bent tail, looped tail, coiled tail and detached tail), as described by Alkan et al. [49].

### 2.8. Statistical Analyses

Data generated were summarized and expressed as mean ± SD (standard deviation) and presented in tables. Multivariate analysis of variance (MANOVA) was employed to evaluate the effect of the extender and ascorbic acid supplementation, while repeated measures analysis of variance was performed to test the effect of time across the different storage temperatures. A chi-square test was used to test associations between proportions of different sperm morphological defects. Values of *p* < 0.05 were considered statistically significant. The statistical package IBM SPSS version 20 was used for the analysis.

## 3. Results

### 3.1. Baseline Spermiogram of Turkey Toms Used for the Study

The semen characteristics of turkey toms enrolled in the study evaluated before the semen preservation study are presented as the mean ± SD in the Supplementary Material (Table S1). Eighty-eight percent (88.4%) of the ejaculates collected were creamy white in color (not presented in the table). A photomicrograph of fresh undiluted turkey semen stained with eosin-nigrosin is presented in the Supplementary Material (Figure S1).

### 3.2. Quality of Preserved Turkey Semen

Generally, there was no significant variation between the two extenders with regard to semen quality (*p* = 0.310 Wilks’ lambda test), while ascorbic acid supplementation had a significant effect on quality parameters (*p* = 0.001 Wilks’ lambda test). Similarly, time of storage had a significant effect (*p* = 0.001 Wilks’ lambda test) on the semen quality across the storage temperatures.

#### 3.2.1. Sperm Motility Assessment among Different Semen Extenders

Sperm motility in the extended semen evaluated hourly for seven hours at ambient temperature is presented in Table 1. There was no significant difference (*p* = 0.483) between the egg yolk-based and soybean-based semen extenders. Similarly, Table 2 presents the mean percentage sperm motility of extended and chilled semen evaluated at 6, 12 and 24 h. There was no significant difference (*p* = 0.577) in motility values between the two extenders. However, motility was significantly (*p* = 0.003) higher in ascorbic acid-supplemented groups.

#### 3.2.2. Sperm Viability Assessment among Different Semen Extenders

Sperm viability evaluated hourly for seven hours at ambient temperature is presented in Table 3; there was no significant difference (*p* = 0.904) between the egg yolk-based and soybean-based semen extenders, but there was a significant difference (*p* = 0.030) between ascorbic acid-supplemented and non-supplemented groups. Similarly, in the chilled preserved semen evaluated at 6, 12 and 24 h (Table 4), there was no significant difference (*p* = 0.844) in viability between the two extenders. However, ascorbic acid-supplemented treatments were significantly higher in viability at 6 and 12 h, while at 24 h, all treatments were statistically the same. On the other hand, both motility and viability indices were significantly (*p* = 0.015) higher after 24 h of chilled preservation than after seven hours at ambient temperature.

#### 3.2.3. Sperm Morphology Assessment

The average morphological defects of spermatozoa in the extended semen evaluated hourly for seven hours at ambient temperature and the chilled semen evaluated at 6, 12 and 24 h are presented in Table 5 and Table 6, respectively. There was no significant difference (*p* = 0.094) between the egg yolk-based and soybean-based semen extenders. However, the ascorbic acid-supplemented groups presented significantly (*p* = 0.001) lower values.

Table 7 presents the proportion of different morphological abnormalities in fresh, chilled and ambient temperature-preserved samples. Mid-piece defects were significantly higher (*p* = 0.001) in fresh semen than in the treated semen, while tail defects increased in preserved semen.

## 4. Discussion

The current study showed that soybean- and egg yolk-based extenders were able to preserve, both at ambient and chilling temperatures, the quality parameters of turkey semen, while there was a significant reduction in turkey sperm morphological defects through the period of preservation when the extenders were supplemented with L-ascorbic acid. Additionally, to the best of our knowledge, this is the first time a study describing the effect of egg yolk plasma and soybean on the liquid storage of turkey semen has been reported.

A baseline spermiogram from the fresh semen of turkey toms was established in the present study. It was quite similar to other reported semen characteristics of turkey toms found in other parts of Nigeria [50,51,52]. However, slightly higher values were reported in exotic breeds [41,45,50,51,53].

In this study, higher motility and viability indices were recorded 24 h after preservation at 4 °C compared with the values recorded after 7 h at ambient temperature. This delineated the importance of a low temperature for liquid preservation of turkey semen [22,54]. However, there was a decrease in motility during the 7 h of holding time at ambient temperature and through the 24 h of chilled storage in all the extender preparations, as reported in crane semen [12,14,15,55]. This is conceivable because at ambient temperature, spermatozoan metabolism is at its peak with a resultant buildup of metabolic byproducts which could slow down sperm motility.

In the current study, there was no difference in semen quality parameters between egg yolk-based and soybean-based extenders, which means soybean is similar to egg yolk and can replace it in preservation media of turkey semen. The survival of turkey spermatozoa in these extenders could be attributed to the neutralization effect of LDL on seminal plasma proteins and other macromolecules that are found in turkey semen [30]. Although the use of whole egg yolk as a supplement in poultry extenders has been associated with reduced fertility, it could be associated with the inadvertent inclusion of the inner perivitelline membrane of egg yolk in the yolk homogenate during yolk collection. The inner perivitelline membrane has been associated with in vitro activation of acrosomal activity of avian sperm [56,57]. Thus, in order to mitigate such an occurrence in this study, we isolated the egg yolk plasma fraction which contained mainly LDL.

Success with the use of soybean-based extenders has been achieved in many animal species [34,35,58,59,60]. Significantly higher post-thaw bovine sperm motility in a soybean lecithin-based extender than Tris-egg yolk diluents has been reported [59]. Similarly, Singh et al. [42] observed that a 25% soy-based extender produced a better sperm quality in chilled bovine semen. On the other hand, no significant difference was observed in post-thaw semen quality when 1% soy lecithin was compared with 20% egg yolk supplementation [61]. It should be highlighted that the aforementioned comparable results between egg yolk- and soybean-based extenders were obtained with a low concentration of soymilk (1%). Similarly, in this study, we reported a comparable result between 1% soybean and 5% egg yolk plasma. This echoes similar findings on roosters and some mammalian species [31,34,35,59,60]. Nonetheless, Singh et al. [42] reported that sperm quality was negatively affected when the soybean milk concentration was greater than 30%. Several studies using a wide range of soybean milk concentrations found wide variation in the results, and the varying degrees of success showed the lack of standardization in the use of soybean-based extenders [31,34,35,42,59,60,61]. This could be associated with the sperm plasmalemma or the seminal plasma composition between species which may determine the required concentration of soybean milk to be used in extenders [33].

The semen viability index in both preservation conditions exhibited a similar pattern to that of motility. However, viability was sustained even when the motility was almost zero. This indicates that motility is the most sensitive parameter to external factors, as also reported with Bestville poultry semen extenders [54]. Sexton [14] also found that storing turkey semen diluted (1:2) with Lake’s solution at 25 °C or 5 °C for 30 min had a profound negative effect on motility, but little effect on the viability index.

In the current report, there was a significant difference between non-supplemented and ascorbic acid-supplemented extenders, with higher values recorded in the latter. This echoes earlier findings where the quality parameters of rooster semen were improved with ascorbic acid supplementation [39,62,63]. However, this result is in contrast to previous submissions by Donoghue and Donoghue [54]. In some reports, ascorbic acid exerts no significant effect on sperm motility but exhibits a profound positive effect on sperm viability [64]. Thus, the positive effect of ascorbic acid supplementation recorded in this study could be associated with the low concentration used. Ascorbic acid alone or in combination with other antioxidants may function as an antioxidant at lower concentrations; otherwise, it may serve as a pro-oxidant, leading to a massive buildup of reactive oxygen species [65]. Its function as an antioxidant could also depend on the composition of the extension media and the concentration used.

A proportional increase in morphologically abnormal spermatozoa was evident in all the preservation protocols and for all the extender preparations. This could be attributed to cellular damage due to the accumulation of metabolic byproducts, especially ROS [12,55,66]. Although there was no significant difference in the morphological abnormality index between egg yolk-based and soybean-based semen extenders, a significantly lower abnormality index was recorded in the ascorbic acid-supplemented fraction of both extenders. This shows the protective effect of ascorbic acid against the harmful effects of lipid peroxidation during storage, as reported previously [62,63].

This study also found a significantly higher proportion of mid-piece defects in fresh semen than in the other treatments. Alkan et al. [49] also reported that acrosomal and mid-piece defects where the most prevalent in the semen of American Bronze turkeys. In other avian species such as the emu, a similar submission was reported on their fresh semen [67]. Recently, Łukaszewicz et al. [68], working with Muscovy ducks, reported findings in line with those in this study regarding mid-piece defects. The mid-piece is the area that harbors all the spermatozoal mitochondria, which has been attributed to ROS generation through leakages in the electron transfer chain [15,66,69,70]. This finding echoes another report where it was clearly demonstrated that ROS generation in mitochondria has a direct causal relationship with mid-piece defects [71]. However, in extended semen, tail defects predominate in both preservation protocols. Siudzińska and Łukaszewicz [72] reported on some chicken breeds and stated that tail defects could be attributed to osmotic variations associated with extended semen.

## 5. Conclusions

It can be concluded that the two extenders (egg yolk-based and soybean-based) were similar with regard to semen quality parameters, and the two preserved turkey semen for up to 24 h of chilled liquid storage. It can also be concluded that ascorbic acid supplementation of the turkey semen extenders improved semen quality parameters including motility and viability and reduced the rate of morphological defects during liquid storage. Thus, the two extenders supplemented with L-ascorbic acid can be used to preserve turkey semen. It is obvious, however, that more studies are required to evaluate semen quality parameters using our extenders in comparison with commercial or other extenders that are already used in turkeys vis à vis fertility and hatchability. It is also pertinent to further study and optimize the level of ascorbic acid supplementation as well as exploring the effect of other antioxidants.

## Figures and Tables

**Table 1:** Motility (%) of turkey spermatozoa during 7 h of storage at ambient temperature.

Treatment Hroups	Time (Hours)							
	0	1	2	3	4	5	6	7
Egg yolk based (EYR)	84.0 ± 8.4 ^a^	74.0 ± 8.4 ^a^	69.5 ± 7.6 ^a^	54.0 ± 13.5 ^a^	43.0 ± 13.4 ^a^	26.0 ± 19.6 ^a^	16.6 ± 19.0 ^a^	7.0 ± 13.4 ^a^
Soybean based (SBR)	81.2 ± 5.2 ^a^	73.5 ± 8.2 ^a^	67.0 ± 9.8 ^a^	54.0 ± 10.8 ^a^	43.0 ± 11.6 ^a^	29.0 ± 12.9 ^a^	17.0 ± 10.6 ^a^	4.0 ± 7.0 ^a^
EYR + L-ascorbic acid	87.1 ± 5.1^b^	80.0 ± 9.4 ^b^	71.0 ± 9.9 ^a^	59.0 ± 4.0 ^a^	47.0 ± 14.9 ^a^	35.0 ± 16.5 ^a^	22.0 ± 14.0 ^a^	11.0 ± 8.8 ^a^
SBR + L-ascorbic acid	86.8 ± 7.4 ^b^	80.0 ± 8.2 ^b^	68.0 ± 9.2 ^a^	54.0 ± 11.7 ^a^	43.0 ± 15.7 ^a^	31.0 ± 17.3 ^a^	17.0 ± 13.4 ^a^	7.0 ± 9.5 ^a^

The data provided in the table are percentages (%) of the mean motility ± SD of the pooled turkey ejaculates included in the study. Values in the same column with different superscripts differ significantly (*p* < 0.05).

**Table 2:** Motility (%) of turkey spermatozoa over 24 h at 4 °C.

Treatment Groups	Time (Hours)		
	6	12	24
Egg yolk based (EYR)	64.0 ± 9.7 ^a^	45.0 ± 15.1^a^	30.5 ± 14.6 ^a^
Soybean based (SBR)	61.0 ± 12.9 ^a^	46.0 ± 17.8 ^a^	29.0 ± 20.4 ^a^
EYR + L-ascorbic acid	77.5 ± 4.3 ^b^	64.0 ± 5.2 ^b^	47.0 ± 7.5 ^b^
SBR + L-ascorbic acid	76.5 ± 4.7 ^b^	63.0 ± 8.2 ^b^	42.0 ± 12.3 ^b^

The data provided in the table are percentages (%) of the mean motility ± SD of the pooled turkey ejaculates included in the study. Values in the same column with different superscripts differ significantly (*p* < 0.05).

**Table 3:** Viability (%) of turkey spermatozoa during 7 h of storage at ambient temperature.

Treatment Groups	Time (Hours)							
	0	1	2	3	4	5	6	7
Egg yolk based (EYR)	94.0 ± 2.6 ^a^	90.6 ± 2.6 ^a^	85.7 ± 5.3 ^a^	83.0 ± 6.0 ^a^	78.9 ± 6.3 ^a^	74.6 ± 9.1 ^a^	70.4 ± 9.1 ^a^	61.8 ± 16.4 ^a^
Soybean based (SBR)	92.5 ± 3.1 ^a^	88.8 ± 5.5 ^a^	87.2 ± 5.4 ^a^	82.5 ± 5.9 ^a^	80.9 ± 7.5 ^a^	75.7 ± 9.2 ^a^	70.9 ± 14.0 ^a^	63.4 ± 17.8 ^a^
EYR + L-ascorbic acid	99.5 ± 0.9 ^b^	99.3 ± 1.0 ^b^	95.4 ± 3.3 ^b^	91.3 ± 3.2 ^b^	90.3 ± 4.9 ^b^	85.2 ± 6.0 ^b^	79.9 ± 8.6 ^b^	74.7 ± 9.2 ^b^
SBR + L-ascorbic acid	99.4 ± 1.1 ^b^	99.2 ± 0.9 ^b^	95.8 ± 2.7 ^b^	93.3 ± 3.3 ^b^	90.3 ± 3.1 ^b^	85.8 ± 6.2 ^b^	80.9 ± 7.1 ^b^	74.2 ± 5.6 ^b^

The data provided in the table are percentages (%) of the mean viability ± SD of the pooled turkey ejaculates included in the study. Values in the same column with different superscripts differ significantly (*p* < 0.05).

**Table 4:** Viability (%) of turkey spermatozoa over 24 h at 4 °C.

Treatment Groups	Time (Hours)		
	6	12	24
Egg yolk based (EYR)	94.5 ± 2.6 ^a^	90.0 ± 5.7 ^a^	85.6 ± 5.8 ^b^
Soybean based (SBR)	94.9 ± 3.2 ^a^	91.4 ± 4.5 ^a^	89.5 ± 3.9 ^b^
EYR + L-ascorbic acid	97.9 ± 2.5 ^b^	94.0 ± 3.9 ^b^	88.5 ± 5.0 ^b^
SBR + L-ascorbic acid	97.9 ± 2.9 ^b^	94.1 ± 3.4 ^b^	89.7 ± 3.7 ^b^

The data provided in the table are percentages (%) of the mean viability ± SD of the pooled turkey ejaculates included in the study. Values in the same column with different superscripts differ significantly (*p* < 0.05).

**Table 5:** Morphological defects (%) of turkey spermatozoa during 7 h of storage at ambient temperature.

Treatment Groups	Time (Hours)							
	0	1	2	3	4	5	6	7
Egg yolk based (EYR)	7.7 ± 3.7 ^a^	10.2 ± 3.1 ^a^	12.2 ± 4.0 ^a^	17.9 ± 5.7 ^a^	23.6 ± 7.3 ^a^	24.2 ± 6.0 ^a^	26.0 ± 6.1 ^a^	37.2 ± 9.9 ^a^
Soybean based (SBR)	6.9 ± 3.1 ^a^	8.0 ± 2.9 ^b^	9.0 ± 2.7 ^b^	15.9 ± 3.1 ^a^	21.2 ± 7.2 ^a^	23.5 ± 6.3 ^a^	27.2 ± 6.9 ^a^	35.8 ± 8.2 ^a^
EYR + L-ascorbic acid	2.5 ± 1.2 ^b^	4.4 ± 2.0 ^c^	5.8 ± 2.7 ^c^	7.4 ± 1.9 ^b^	9.5 ± 2.7 ^b^	12.2 ± 3.3 ^b^	15.0 ± 3.6 ^b^	17.6 ± 2.7 ^b^
SBR + L-ascorbic acid	2.3 ± 1.2 ^b^	2.6 ± 1.1 ^d^	4.3 ± 1.5 ^d^	6.2 ± 2.3 ^b^	8.4 ± 2.0 ^b^	10.4 ± 2.4 ^b^	12.7 ± 3.4 ^b^	15.2 ± 1.9 ^b^

The data provided in the table are percentages (%) of the mean morphological defects ± SD of the pooled turkey ejaculates included in the study. Values in the same column with different superscripts differ significantly (*p* < 0.05).

**Table 6:** Morphological defects (%) of turkey spermatozoa over 24 h at 4 °C.

Treatment Groups	Time (Hours)		
	6	12	24
Egg yolk based (EYR)	10.2 ± 3.7 ^a^	11.3 ± 2.8 ^a^	16.4 ± 2.2 ^a^
Soybean based (SBR)	7.9 ± 4.6 ^a^	12.8 ± 3.5 ^a^	20.1 ± 5.4 ^b^
EYR + L-ascorbic acid	3.9 ± 1.0 ^b^	7.3 ± 3.1 ^b^	10.2 ± 3.3 ^c^
SBR + L-ascorbic acid	3.6 ± 1.2 ^b^	6.3 ± 2.0 ^b^	9.5 ± 2.1 ^c^

The data provided in the table are percentages (%) of the mean morphological defects ± SD of the pooled turkey ejaculates included in the study. Values in the same column with different superscripts differ significantly (*p* < 0.05).

**Table 7:** Proportions of different sperm morphological defects in different preservation conditions applied to turkey semen.

Preservation	Acrosome	Percentage (%) Head	Mid-Piece	Tail
Fresh semen N = 173	0.0 ^a^	11.8 ^a^	44.4 ^a^	43.8 ^a^
Ambient temperature N = 799	1.5 ^a^	15.4 ^a^	19.2 ^b^	63.8 ^b^
Chilled (4 °C) N = 1399	3.6 ^b^	17.0 ^a^	20.5 ^b^	58.9 ^c^

N = total number of morphological defects. Values in the same column with different superscripts differ significantly (*p* < 0.05).

## Data Availability

Data are contained within the article.

## Supplementary Materials

The following supporting information can be downloaded at: https://www.mdpi.com/article/10.3390/ani12050648/s1, Table S1: Spermiogram of turkey toms enrolled in the study, Figure S1: Photomicrograph of fresh undiluted turkey semen stained with eosin-nigrosin showing live (unstained) and dead sperm (partially stained, arrow).
