# Supplementary material for: Spermatozoa Survival in Egg Yolk-Based and Soybean-Based Extenders at Ambient and Chilling Temperature in Domestic Turkeys (Meleagris gallopavo)
Source: Animals (Basel). 2022 Mar 3;12(5):648. doi: 10.3390/ani12050648 (PMC8909083; doi:10.3390/ani12050648)
Supplement: Supplementary file 1 [file animals-12-00648-s001.zip › animals-1535913-supplementary.pdf]

Table S1. Spermiogram of turkey toms enrolled in the study.

Parameters	Turkey 1<br>N =9	Turkey 2<br>N =16	Turkey 3<br>N =15	Turkey 4<br>N =11	Turkey 5<br>N =11	Turkey 6<br>N =15	Turkey 7<br>N =7	Mean<br>N =84
**Volume (ml)**	0.17 ± 0.14	0.19 ± 0.62	0.19 ± 0.11	0.21 ± 0.11	0.22 ± 0.14	0.35 ± 0.13	0.21 ± 0.11	0.23 ± 0.13<br>(0.20 - 0.25)
**Concentration (x10^9/ml)**	4.18 ± 2.32	4.72 ± 2.42	5.30 ± 2.28	5.46 ± 4.04	4.48 ± 1.84	6.43 ± 2.74	5.07 ± 2.60	5.16 ± 2.66<br>(4.59-5.74)
**Motility (%)**	88.78 ± 5.33	91.50 ± 5.30	86.53 ± 6.00	88.91 ± 9.00	83.72 ± 7.54	85.87 ± 6.31	84.29 ± 9.76	87.22 ± 7.42<br>(85.63 - 88.81)
**Viability (%)**	95.22 ± 3.90	97.06 ± 4.78	97.53 ± 2.19	96.00 ± 4.15	95.73 ± 4.88	96.27 ± 5.27	97.29 ± 2.23	96.44 ± 4.11<br>(95.56 - 97.32)
**Morphological defects (%)**	4.67 ± 3.04	3.44 ± 2.63	5.80 ± 3.55	4.27 ± 2.90	6.46 ± 3.62	4.67 ± 3.31	7.14 ± 3.63	5.54 ± 3.53<br>(4.38 - 5.90)
**pH**	7.29 ± 0.34	7.10 ± 0.34	7.85 ± 0.55	7.73 ± 0.34	7.38 ± 0.48	7.60 ± 0.39	7.57 ± 0.53	7.50 ± 0.49<br>(7.40 - 7.60)

*Values in parenthesis represent the 95% confidence interval for each parameter. N= number of replications.

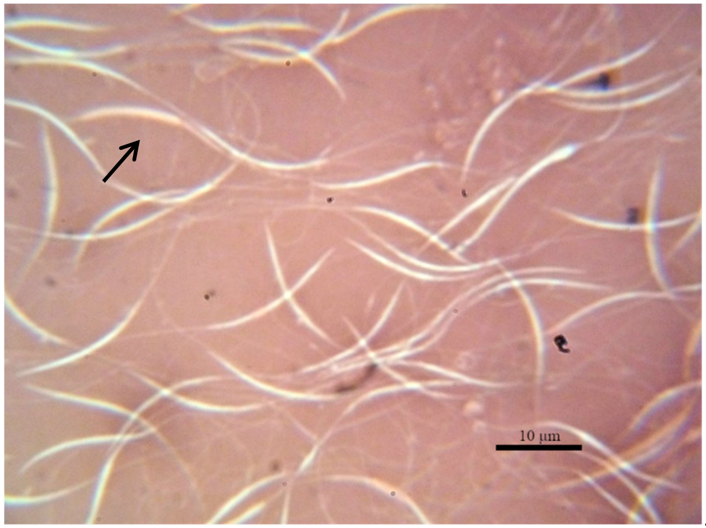

Figure S1. Photomicrograph of fresh undiluted turkey semen stained with eosin-nigrosin showing live (unstained) and dead sperm (partially stained, arrow).
